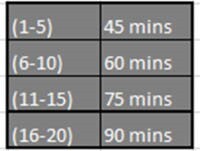# 727 Development of an Acuity and Scheduling System for Wound Care

**DOI:** 10.1093/jbcr/irad045.202

**Published:** 2023-05-15

**Authors:** Elizabeth Salazar, Alina Ruiz, Amber Ewing, Michelle Terry

**Affiliations:** Parkland Health, Garland, Texas; Parkland Health, Garland, Texas; Parkland, Fate, Texas; Parkland Health, Frisco, Texas; Parkland Health, Garland, Texas; Parkland Health, Garland, Texas; Parkland, Fate, Texas; Parkland Health, Frisco, Texas; Parkland Health, Garland, Texas; Parkland Health, Garland, Texas; Parkland, Fate, Texas; Parkland Health, Frisco, Texas; Parkland Health, Garland, Texas; Parkland Health, Garland, Texas; Parkland, Fate, Texas; Parkland Health, Frisco, Texas

## Abstract

**Introduction:**

Acuity systems have been used in nursing to quantify the care needed for each patient based on the patient’s nursing needs. The benefits for using an acuity system include avoiding over or understaffing and improving patient outcomes. To date no formal acuity system has been used to quantify the work associated with burn wound care. The purpose of this quality assurance project was to develop an acuity and scheduling system for burn patients and provide more effective and equitable staffing for burn wound care.

**Methods:**

A wound care subgroup was formed by Unit Based Council to develop a point-based acuity scale for wound care. Numerical points were assigned for each body area dressed and wound care procedures such as surgical dressing take downs, moderate sedation, staple removal, wound vac change, wraps or casting, initial debridement, and discharge teaching (Fig. 1). Points were totaled and associated with a range of time: 1-5 points equals 45 minutes; 6-10 points equals 60 minutes; 11-15 points equals 75 minutes, and 16-20 points equals 90 minutes (Fig. 2). The total time of all dressing changes for the day based on the acuity system determines the RN and Tech staffing for wound care. After considering provider and therapy priorities, a schedule is posted in Microsoft Teams allowing for interdisciplinary collaboration and visibility on wound care scheduling.

**Results:**

To date the acuity-based schedule has been used to schedule 500 consecutive dressings. Acuity based scheduling has helped to quantify the number of staff needed for wound care related to the amount of time needed each day. Prior to the initiation of this practice, wound care was assigned based on the number of patients instead of the workload required per patient leading to over or understaffing. The use of the acuity system has allowed the unit to predict the number of staff needed for wound care each day and adherence to the schedule with a margin of 30 minutes plus/minus was achieved.

**Conclusions:**

Use of an acuity and scheduling system for burn wound care can improve staffing and will be a tool to quantify productivity.

**Applicability of Research to Practice:**

Acuity systems and wound care scheduling can improve staffing for burn centers.